# Promise(s) of Mesenchymal Stem Cells as an *In Vitro* Model System to Depict Pre-Diabetic/Diabetic Milieu in WNIN/GR-Ob Mutant Rats

**DOI:** 10.1371/journal.pone.0048061

**Published:** 2012-10-29

**Authors:** Soundarya L. Madhira, Satya S. Challa, Maniprabha Chalasani, Giridharan Nappanveethl, Ramesh R. Bhonde, Rajanna Ajumeera, Vijayalakshmi Venkatesan

**Affiliations:** 1 Department of Biochemistry/Stem Cell Research, National Institute of Nutrition, Hyderabad, Andhra Pradesh, India; 2 National Centre for Laboratory Animal Sciences, National Institute of Nutrition, Hyderabad, Andhra Pradesh, India; 3 Manipal Institute of Regenerative Medicine, Bangalore, Karnataka, India; Children’s Hospital Boston, United States of America

## Abstract

**Background:**

Development of model systems have helped to a large extent, in bridging gap to understand the mechanism(s) of disease including diabetes. Interestingly, WNIN/GR-Ob rats (Mutants), established at National Centre for Laboratory Animals (NCLAS) of National Institute of Nutrition (NIN), form a suitable model system to study obesity with Type 2 diabetes (T2D) demonstrating several secondary complications (cataract, cardiovascular complications, infertility, nephropathy etc). The present study has been carried out to explore the potent application(s) of multipotent stem cells such as bone marrow mesenchymal stem cells (BM-MSCs), to portray features of pre-diabetic/T2D vis-à-vis featuring obesity, with impaired glucose tolerance (IGT), hyperinsulinemia (HI) and insulin resistance (IR) seen with Mutant rats akin to human situation.

**Methodology/Principal Findings:**

Primary cultures of BM-MSCs (third passage) from Mutants, its lean littermate (Lean) and parental control (Control) were characterized for: proliferation markers, disease memory to mark obesity/T2D/HI/IR which included phased gene expression studies for adipogenic/pancreatic lineages, inflammatory markers and differentiation ability to form mature adipocytes/Insulin-like cellular aggregates (ILCAs). The data showed that BM-MSCs from Mutant demonstrated a state of disease memory, depicted by an upregulated expression of inflammatory markers (IL-6, TNFα); increased stem cell recruitment (Oct-4, Sox-2) and proliferation rates (CD90+/CD29+, PDA, ‘S’ phase of cell cycle by FACS and BrdU incorporation); accelerated preadipocyte induction (Dact-1, PPARγ2) with a quantitative increase in mature adipocyte formation (Leptin); ILCAs, which were non-responsive to high glucose did confer the Obese/T2D memory in Mutants. Further, these observations were in compliance with the anthropometric data.

**Conclusions:**

Given the ease of accessibility and availability of MSCs, the present study form the basis to report for the first time, application of BM-MSCs as a feasible *in vitro* model system to portray the disease memory of pre-clinical/T2D with IR - a major metabolic disorder of global concern.

## Introduction

Diabetes mellitus is a debilitating metabolic syndrome arising either due to (i) autoimmune destruction of the pancreatic beta cells (β-cells) resulting in absolute insulin deficiency (Type 1 diabetes (T1D)), or (ii) impaired glucose uptake attributed to peripheral resistance in target organs such as muscle and adipose tissue, as well as (iii) β-cell exhaustation in chronic conditions (Type 2 diabetes (T2D)). More recent studies document diabetes as a state of profound and consistent oxidative stress/chronic inflammatory condition [1] evidenced by increased levels of free radicals [1], [2], with a concomitant decrease in antioxidant status *in situ* [3]. Interestingly, compared to all other organs/cells in the body, β-cells of pancreas are highly vulnerable to oxidative damage on account of its inherently weak antioxidant systems [4] causing impaired β-cell functions, resulting in an imbalance of glucose homeostasis vis-à-vis altered cellular microenvironment.

Cellular microenvironment/niche is a critical unit, patterned to orchestrate the integration of intrinsic factors with extrinsic cues in a spatiotemporal manner for the maintenance of cellular integrity and metabolism [5]. In addition, cells also rely on a variety of potent antioxidant defense mechanisms [6], and a close interaction of antioxidant molecules generated in actively growing and proliferating cells help to scavenge the reactive oxygen species [7]. This is significant in view of the recent findings to support diabetes as a state of oxidative stress [8], affecting various soluble and insoluble factors that form part of microenvironment/niche, affecting the stem cell pool.

Mesenchymal stem cells (MSCs) are a promising source of adult stem cells, well documented for their plasticity and differentiation potential to form multiple cell types *in vitro* and *in vivo* [9]. Recent studies have documented the immunomodulatory functions (i.e. secretion of various angiogenic and cytoprotective factors) [10], [11] and their potential to improve healthcare by either augmenting body’s own regenerative potential or development of new therapies [12]. A dysregulation in the microenvironment of MSCs from human subjects have shown for an altered surface marker expression, self-renewal capacities in several pathological conditions such as chronic renal failure [13], multiple myeloma [14], acute myeloid leukemia [15], rheumatoid arthritis [16] and T1D [17] and T2D [18], demonstrating upregulated expression of inflammatory cytokines for depicting the disease memory towards understanding the cellular and molecular basis of the alterations.

In the present study, we have investigated BM-MSCs (multipotent stem cells) to assess for the altered cellular milieu in WNIN/GR-Ob (Mutant) rats which depict obesity, impaired glucose tolerance (IGT) with insulin resistance (IR) similar to the preclinical/clinical features of T2D seen in human subjects. To address these, we have calculated IR status, assessed for mesenchymal phenotype and proliferation rate, studied the gene expression profile in BM-MSCs to mark the disease memory depicting obesity, IGT with IR including the inflammatory status, and finally we have examined for differentiation/functional capabilities of BM-MSCs to form mature adipocytes and Insulin-like cellular aggregates (ILCAs). All these parameters have been compared between Mutants, their lean littermates (Lean) and parental controls (Control) under the same experimental conditions.

## Results

### Anthropometric Measurements

Body weights were significantly higher in Mutant (450±32.8 gms*/p = 0.03), compared to Lean (250.6±19.9 gms) and Control (290.2±20.8 gms). A concomitant increase in the adipose depot weights among Mutant (23.92±4.85 gms*/p = 0.04) was also significant compared to Lean (15.05±3.1 gms) and Control (16.41±2.9 gms). Values have been represented as Mean ± Standard error (SE) from six animals per group.

### Blood and Plasma Measurements

Published reports from these Mutants have shown a normoglycemic response with fasting blood glucose (FBG) in the range of 50–109 mg/dl [19]. In similar lines, we have also demonstrated normoglycemia in Mutant (91.0±2.83 mg/dl), Lean (74.5±4.95 mg/dl) and Control (77.5±2.12 mg/dl). Measurement of fasting plasma insulin (FPI) by ELISA [20], showed a hyperinsulinemic response which was significantly increased in Mutant (21.76±5.76* µU/ml, p = 0.01) compared to Lean (7.24±2.05 µU/ml) and Control (3.63±2.45 µU/ml). Using these FBG and FPI values, indices of IR - Homeostasis Model of Assessment for Insulin Resistance (HOMA-IR), Fasting Glucose to Insulin Ratio (FGIR) and Quantitative Insulin Sensitivity Check Index (QUICKI) were calculated for Mutant, Lean and Control using a mathematical model published earlier [21]. HOMA-IR was significantly increased, while FGIR and QUICKI indices were significantly decreased among Mutant compared to Lean and Control, indicating a state of IR in Mutants (Table 1). All measurements have been represented as Mean ± SE (n = 6 per group).

**Table 1:** Blood and Plasma measurements from WNIN/GR-Ob Rats.

Parameter	Mutant	Lean	Control
Blood Glucose (mg/dl)	91.0±2.83*	74.5±4.95	77.5±2.12
Plasma Insulin (µU/ml)	21.76±5.76*	7.24±2.05	3.63±2.45
***Indices of Insulin Resistance (IR)***			
FGIR	1.12±0.02*	0.73±0.01*	8.03±0.08
QUICKI	0.01±0.0*	0.02±0.0	0.04±0.01
HOMA-IR	1.60±0.04*	1.38±0.02*	0.20±0.01

The Mutant rats demonstrated hyperinsulinemia which was significant compared to their Lean and Control. An increase in blood glucose levels was also evident among Mutants. IR was calculated based on three functions - a) FGIR <4.5 mg/10^−4^U; b) QUICKI – Lower among Obese; c) HOMA-IR >1 Asteric (*) indicates significance at p<0.05 by ANOVA compared to control. Values are represented as Mean ± SE (n = 6) from six animals per group.

Abbreviations:

FGIR: Fasting Glucose to Insulin Ratio, QUICKI: Quantitative Insulin Sensitivity Check Index, HOMA-IR: Homeostasis Model of Assessment for Insulin Resistance.

### Characterization of MSCs during *in vitro* Expansion

**Figure 1:**
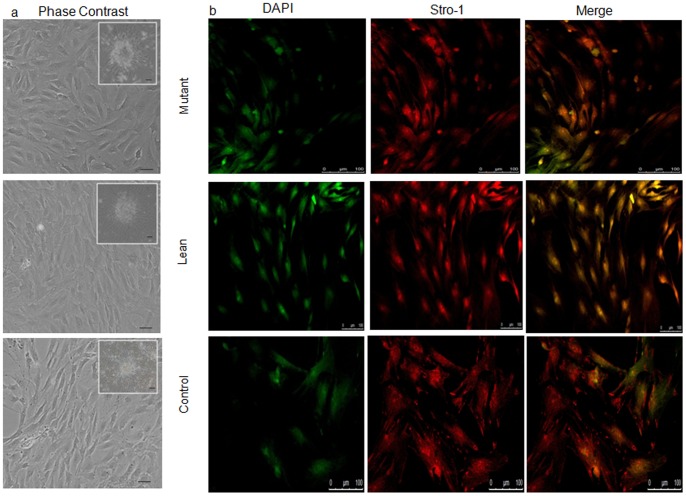
Characterization of the primary cultures of BM-MSCs. (A) Phase contrast photomicrographs showing primary cultures of BM-MSCs (day 7) from Mutant, Lean and Control. Spindle-like cell morphology with colony-formation units (CFU) were seen (insight), which have been captured using ACT2U software attached to Nikon Microscope at magnifications represented using scale bar. (B) MSCs characterized for mesenchymal-specific cytosolic protein STRO-1 (red) and cell nuclei were stained with 4′,6-diamidino-2-phenylindole (DAPI) (green - pseudo color). Images were captured in Confocal Microscope using Leica Advanced Fluorescence software (Leica SP5 series, Germany) at magnifications represented using scale bars.

**Figure 2:**
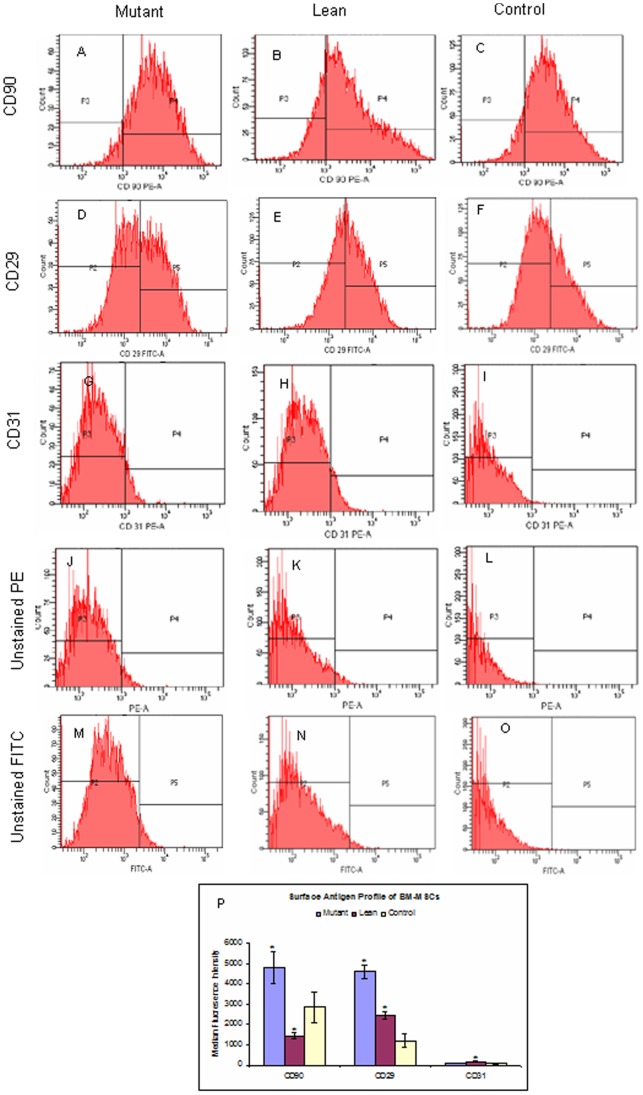
Flow Cytometric analysis of BM-MSCs. Representative FACS analyses (FACS Diva software, FACS Aria II, BD, CA) of MSCs at passage 3 were positive for CD90-PE (A, B & C) and CD29-FITC (D, E & F) and negative for CD31-PE (G, H & I) demonstrating a mesenchymal phenotype from Mutant, Lean and Control respectively. Panels J, K & L and M, N & O represent the negative controls for PE (CD90/CD31) and FITC (CD29) fluorochromes respectively. Median Fluorescence Intensity (MFI) for CD90, CD29 and CD31 have been indicated in panel P and values have been represented as Mean ± SE (n = 6) from three independent experiments performed in duplicates for each phenotype. Values represented have been normalized against unstained controls for the fluorophore (PE/FITC).

MSCs were seeded as single cells onto DMEM/F12+10% fetal bovine serum (FBS). By 2–5 days after isolation, spindle shaped adherent cells were observed. Non-adherent cells were removed during subsequent media changes. These adherent cells formed a monolayer by day 7 (Figure 1A) and also showed the colony forming unit (CFU) featuring an MSC phenotype (insight in Figure 1A). Such confluent cultures largely comprised of MSCs which were positive for mesenchymal stem cell-specific cytosolic protein STRO-1 (Figure 1B). Homogeneity of the population was further confirmed by characterization of the surface phenotype by flow Cytometry (FACS) and represented as median fluorescence intensity (MFI) as given in Figure 2P. Expression of mesenchymal specific surface proteins such as CD90 was higher in Mutant (4798±787 p = 0.02) (Figure 2A), when compared to Lean (1431±153) (Figure 2B) and Control (2872±763) (Figure 2C) although, the expression of CD90 from Lean was of lesser magnitude than that from Control. A similar significant increase in expression of CD29 among Mutant (4619±314) (Figure 2D) compared to Lean (2434±184, p = 0.02) (Figure 2E) and Control (1178±326) was also evident (Figure 2F). This shows that the differential expression levels of both the surface markers (CD90 & CD29) showed a slight difference among the groups.

Interestingly, CD31 which was used as a negative marker for MSCs was almost absent in Control (61±19) (Figure 2I) as compared to Mutant (82±16, p = 0.04) (Figure 2G) and Lean (163±14, p = 0.02) (Figure 2H) which was significantly high. Figures 2J–L and 2M–O show the negative controls run parallely during the analysis for CD90/CD31 and CD29 respectively.

### Proliferation Rate

#### A) Population doubling assay (PDA)

**Figure 3:**
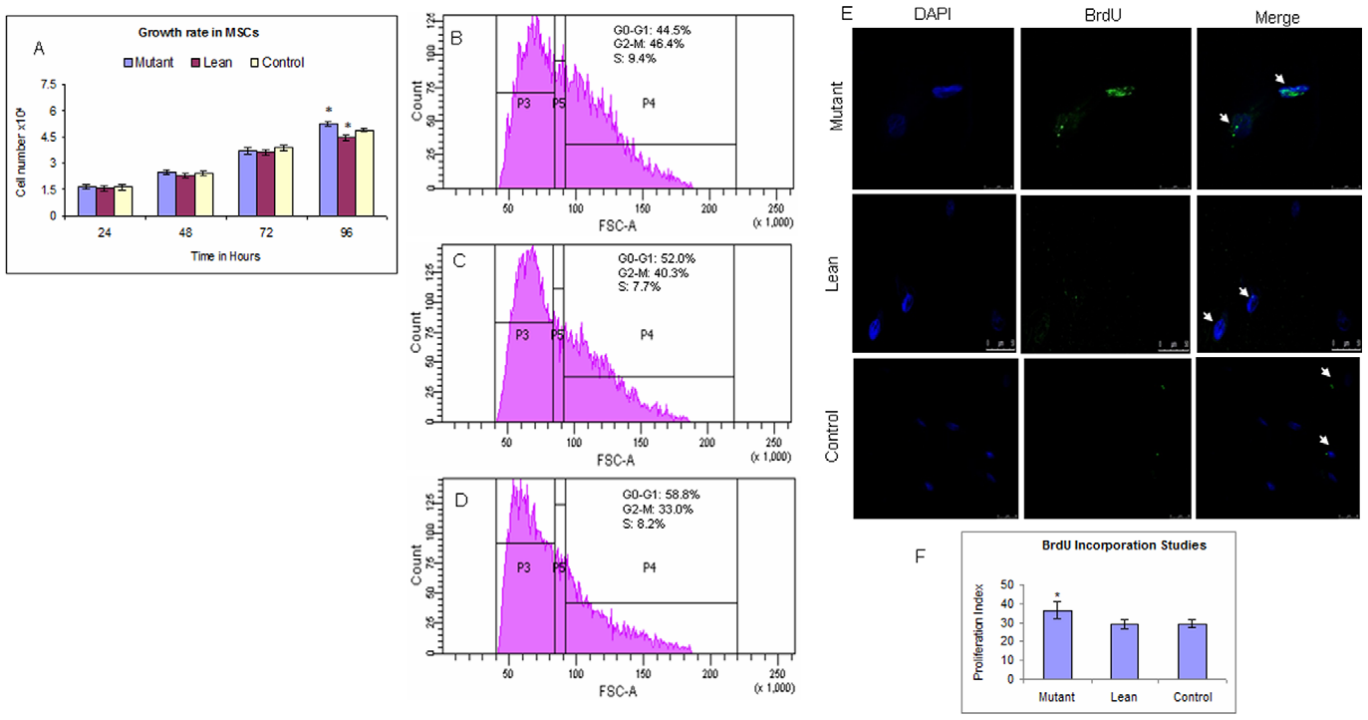
Population doubling assay and Cell cycle analysis of BM-MSCs. (A) MSCs from Mutant, Lean and Control, were measured for proliferation rate at 24, 48, 72 and 96 hrs. BM-MSCs from Mutants showed an increase in proliferation at all the time points and at 96 hours, the PDA was significantly higher compared to Lean and Control. Values represent the absolute cell count and have been computed from three independent experiments performed in duplicates (Mean ± SE, n = 6) (B) Cell cycle analysis of BM-MSCs from Mutants showing an increase in ‘S’ phase and G2-M as compared to Lean (C) and Control (D). Values have been represented as Mean ± SE (n = 6) from three individual experiments performed in duplicates. (E) Cell cycle analysis of BM-MSCs showing an increase in BrdU incorporation (‘S’ phase) (green) from Mutant compared to Lean and Control and proliferation index (F). Values have been represented as Mean ± SE (n = 6) from three individual experiments performed in duplicates. Asteric (*) indicates p<0.05 using ANOVA.

MSCs at passage 3 were subjected to PDA as per our earlier published protocol [22]. MSCs from Mutant showed an increase in proliferation rates at all the time points (24, 48, and 72 hrs) compared to Lean and Control. However at 96 hrs, PDA was statistically significant in Mutant (5.27±0.13×10^4^, p = 0.04) when compared to Lean (4.47±0.16×10^4^) and Control (4.90±0.11×10^4^) (Figure 3A). Values represent an average of three independent experiments each performed in duplicates (Mean ± SE).

#### B) Cell cycle analysis

Cell cycle analysis, demonstrated a significant increase in synthetic (‘S’) phase from BM-MSCs of Mutant (9.4±1.1%, p = 0.03) (Figure 3B) compared to Lean (7.7±0.4%) (Figure 3C) and Control (8.2±0.2%) (Figure 3D), in agreement with our results obtained from PDA (Figure 3A). In similar lines, percentage of population in S+G2-M phase was also significantly higher among Mutant (54.8±2.5%, p = 0.01) as compared to Lean (48.0±3.1%) and Control (41.2±2.8%) (Figure 3B–D) Values given are representative data of Mean ± SE (n = per group) of three independent experiments performed in duplicates.

#### C) Bromodeoxy Uridine (BrdU) labelling studies

Proliferation index showed an increase in BrdU incorporation (proliferation index) from BM-MSCs of Mutant (36.65±4.5%, p = 0.04) compared to Lean (29.15±2.6%) and Control (29.43±3.2%) (Figure 3E&F), which is in agreement with our results obtained from PDA and cell cycle analysis by FACS (Figure 3A–D). Values given are representative data of Mean ± SE (n = 6 per group) of three independent experiments performed in duplicates.

### Gene Expression by RT-PCR

#### For Adipocyte lineage

**Figure 4:**
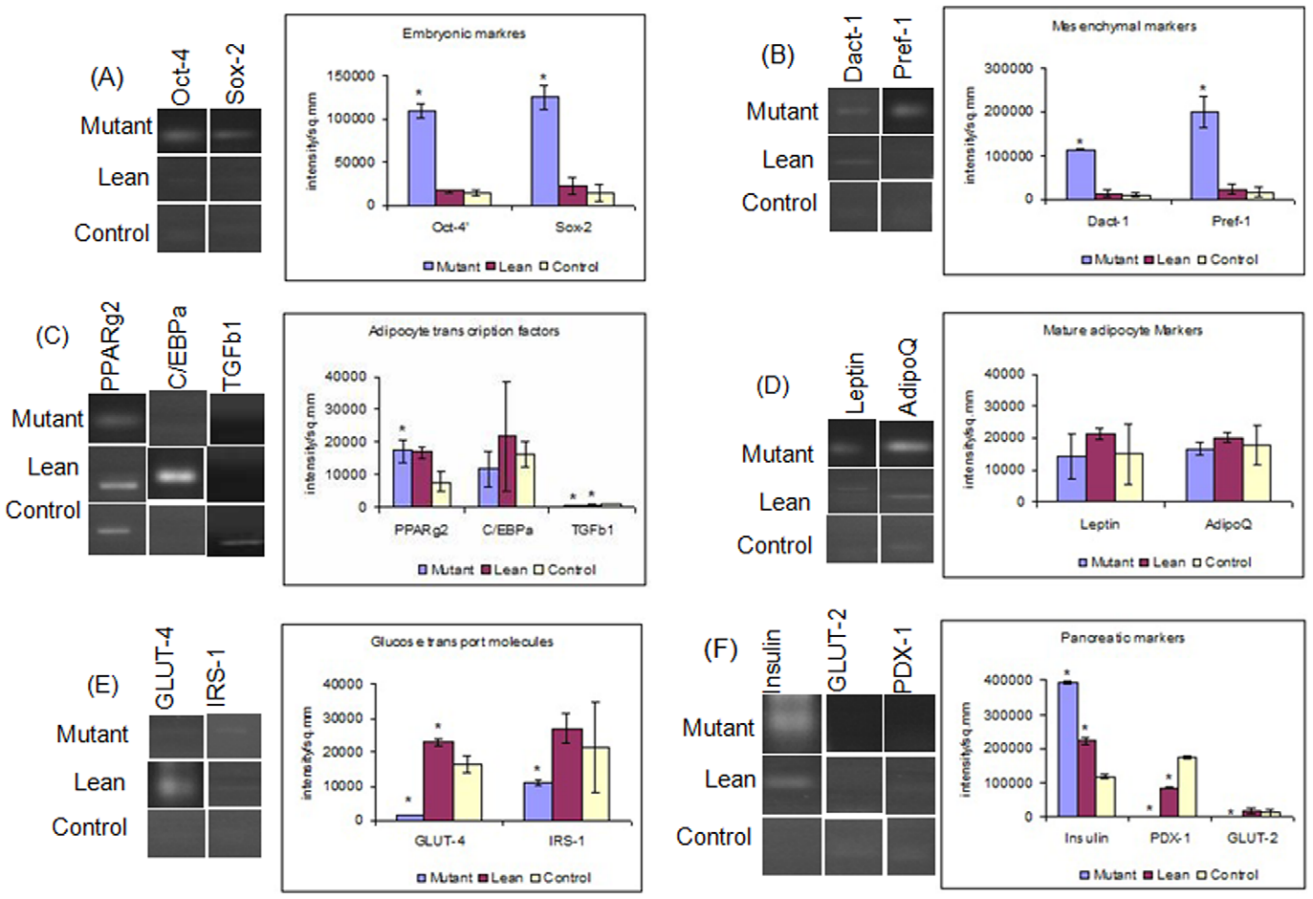
Gene expression analysis of BM-MSCs. BM-MSCs from Mutants demonstrated an upregulated expression of A) Embryonic (Oct-4/Sox-2 (Embryonic markers), (B) Mesenchymal/Preadipocyte markers (Dact-1/Pref-1) and (C) transcriptional factor (PPARγ2) compared to Lean and Control. However, TGFβ1 showed a decrease (C) and C/EBPα remained the same (C). Mature adipocyte markers Leptin and adiponectin (D) and GLUT-4 and IRS-1 (glucose homeostasis markers) (E) were decreased in Mutant. An upregulated expression of Insulin in Mutant, while PDX-1 and GLUT-2 levels were decreased in Mutant as compared to Lean and Control (F). Quantitative measurements of gene expression were carried out by densitometric analyses using QuantityOne software (BioRad, Italy) and represented graphically by plotting intensity/sq.mm vs gene/phenotype. Values represent Mean ± SE (n = 6) from three independent experiments performed in duplicates. Asteric (*) represents significance (p<0.05 by ANOVA) compared to Control.

mRNA expression for embryonic markers such as Oct-4 and Sox-2 showed an upregulation among MSCs from Mutant phenotype (p = 0.001) compared to its Lean and Control (Figure 4A). A similar increase was also noted in Mutants with other markers such as Dact-1 (mesenchymal marker), preadipocyte factor 1 (Pref-1, a preadipocyte marker) (Figure 4B) and peroxisome proliferator activator receptor gamma 2 (PPARγ2) (a master adipocyte transcription factor for adipogenesis), compared to Control (Mutant∼ Lean> Control) as given in Figure 4C. The transcript levels of mature adipocyte markers such as Leptin, Adiponectin (Figure 4D) and CCAAT/enhancer- binding protein alpha (C/EBPα) (adipocyte transcription factor) (Figure 4C) were decreased in Mutant and Control compared to Lean. However, the difference in these expression levels was not statistically significant. In addition, transcript levels of transforming growth factor beta 1 (TGFβ1) involved in osteogenesis was significantly decreased (p = 0.02) among Mutant compared to Lean and Control (Figure 4C). Interestingly, expression of glucose transporter-4 (GLUT-4) and Insulin receptor substrate 1 (IRS-1), which primarily participate in peripheral glucose uptake, were significantly decreased (p = 0.001) in Mutant implicating for an impaired glucose uptake in these Mutants compared to Lean and Control (Figure 4E).

#### For pancreatic lineage

Since these Mutants demonstrate obesity with IGT and IR, we assessed mRNA expression levels of key genes of the β-cell lineage in BM-MSCs amongst the three groups. Interestingly, BM-MSCs from Mutant and Lean showed significantly upregulated expression of Insulin (Mutant>Lean) compared to Control (p = 0.02). On the other hand, expression of both pancreatic duodenum homeobox-1 (PDX-1) - a pancreatic transcriptional factor and glucose transporter-2 (GLUT-2) - a pancreatic glucose transporter were significantly decreased in BM-MSCs of Mutant compared to Lean and Control suggesting for an IGT in Mutants (Figure 4F).

### Inflammatory and Stress Markers

Thiobarbituric acid reacting species (TBARS) as a measure of global oxidative stress was measured in plasma (levels in circulation) from Mutant, Lean and Control. Levels of Plasma TBARS were found to be significantly higher in Mutant (2.70±0.59* nM/ml) compared to Lean (2.59±0.28 nM/ml) and Controls (2.15±0.34 nM/ml).

**Figure 5:**
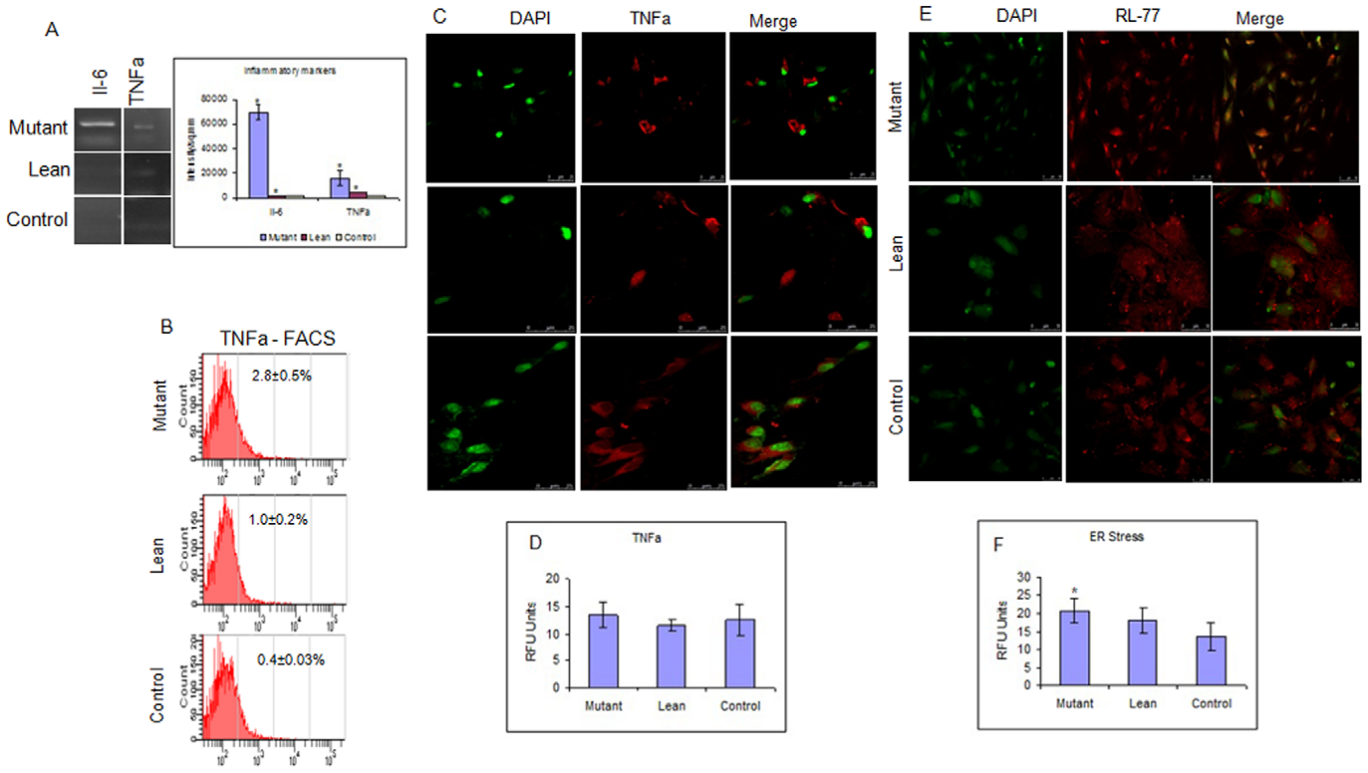
Assessment of Stress responses among BM-MSCs. (A) Increased expression of Interleukin-6 (IL-6) and Tumor necrosis factor alpha (TNFα) (inflammatory markers) was noted in MSCs from Mutant compared to Lean and Control. FACS analysis also showed an increase in TNFα in Mutant compared to Lean and Control (B). Immunolocalization data for TNFα (red) (C) and endoplasmic reticulum stress protein RL-77 (red) (E) showed an increase in Mutant compared to Lean and Control. Relative Fluorescence Units (RFU) for TNFα (D) and RL-77 (F) have also been represented. All images were captured at a magnification of 400x and quantified (as RFU) using Leica Advanced Fluorescence software in Leica Confocal Microscope (Leica SP5 series, Germany). Values have been represented as Mean ± SE (n = 6) from three independent experiments performed in duplicates.

mRNA levels of inflammatory cytokines, IL-6 and TNFα were significantly upregulated among BM-MSCs of Mutant, followed by Lean and Control (Figure 5A). Supporting findings such as increased localization of TNFα (Figure 5C) and endoplasmic reticulum (ER) stress protein RL-77 (Figure 5E), further reinstated the inflammatory state in BM-MSCs from Mutant which has also been expressed as relative fluorescence units (RFU) (Figure 5D&F). The increased TNFα immunolocalization among Mutants compared to Lean and Control was however not statistically significant. Confirmatory results by FACS analysis for TNFα (Figure 5B) did support for the increase in *in situ* stress of BM-MSCs from Mutant (2.8±0.5%), compared to Lean (1.0±0.2%) and Control (0.4±0.03%) phenotypes. Data have been compiled from three independent experiments performed in duplicates.

### Differentiation of BM-MSCs to Mature Adipocytes

**Figure 6:**
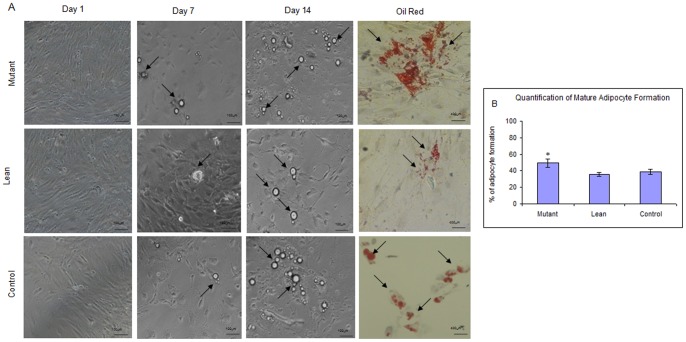
Differentiation of BM-MSCs to form adipocytes. (A) Morphological changes in MSCs from Mutant, Lean and Control rats during adipogenic induction were visualized under phase contrast microscope (Nikon T2000, Japan) at day 1, 7 and 14 demonstrating the formation of lipid-droplet filled cells (arrows). Adipocyte formation was confirmed by staining with Oil Red O (arrows). Magnifications have been represented using scale bar. Panel B shows the quantification for increase in mature adipocytes formed from Mutant as compared to Lean and Control, using the ACT2U soft ware (Nikon, Japan). Values have been represented as Mean ± SE (n = 6), from three independent experiments performed in duplicates. Asteric (*) represents statistical significance (p<0.05) compared to Control as analyzed by ANOVA.

MSCs from Mutant, Lean and Control phenotypes were subjected to adipogenic induction as described earlier [23]. At the end of a 15 day protocol, the oval or near spherical cells filled with lipid droplets that stained positive with oil red (Figure 6A), were quantitated for the mature adipocytes formed using ACT2U software (Nikon, Japan). As indicated in Figure 6B, the number of mature adipocytes formed were much higher from Mutant (49.25±5.6%) compared to Lean (35.46±2.5%) and Control (38.5±3.2%).

### Differentiation of BM-MSCs to ILCAs

**Figure 7:**
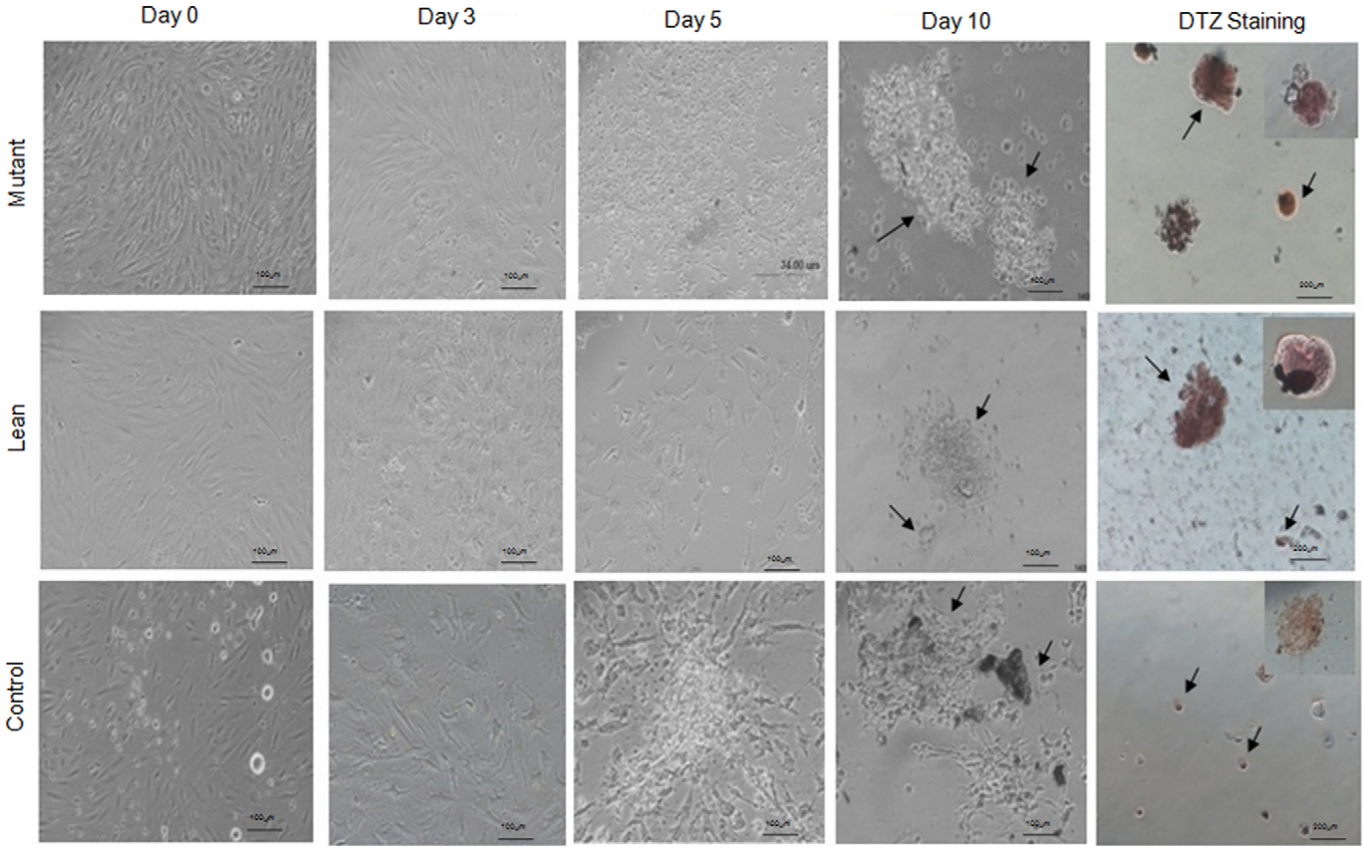
Differentiation of BM-MSCs to form ILCAs. BM-MSCs of Mutant, Lean and Control were subjected to a 10-day differentiation protocol to generate ILCAs. Monolayer of BM-MSCs exposed to serum free medium (SFM)-A (0.5 mM sodium butyrate, 4 nM Activin A and 50 µM β-mercaptoethanol) underwent aggregation by day 3 and when exposed to SFM-B (0.3 mM Taurine) formed clusters/ILCAs by day 5. These ILCAs mature with the addition of SFM-C (3 mM Taurine, 100 nM GLP-1 and 1 mM Nicotinamide) by day 10 and stain deep red/crimson red with dithizone (DTZ) (arrows). The data showed an increase in formation of ILCAs from Mutant compared to Lean and Control. All images were captured in phase contrast microscope (Nikon T2000, Japan) at magnifications, represented using scale bars within the images. Values have been represented as Mean ± SE (n = 6) from three independent experiments performed in duplicates.

MSCs from Mutant, Lean and Control were also studied for the formation of ILCAs using the three-step protocol published earlier [24]. Briefly, addition of serum free medium (SFM)-A initiated the process of differentiation which was evidenced by a change in morphology of MSCs. On addition of SFM-B in the second step, cluster-like formation was evident and maturation of these clusters was appreciable with the addition of SFM-C containing maturation factors such as glucagon-like peptide 1 (GLP-1), Nicotinamide and high concentrations of Taurine (i.e. 3 mM). After 10 days of initiation, the newly formed ILCAs showed deep crimson red color when stained with dithizone (DTZ), which has been used as an islet specific marker and reflects maturation of the clusters. Amongst the three groups, BM-MSCs from Mutant showed an increased formation of ILCAs compared to Lean and Control (Figure 7).

ILCAs obtained from Mutant, Lean and Control were next measured for their insulin secretory response at both basal (5.5 mM) and high glucose challenge (17.5 mM) similar to our published data [20]. The fact that these Mutants were hyperinsulinemic was evident by their higher basal insulin levels in Mutant (50.71±0.13 µU/L/mg protein) and Lean (42.23±0.15 µU/L/mg protein) compared to Control (41.11±0.09 µU/L/mg protein) (Table 2) and these ILCAs were less responsive to glucose challenge from basal to high glucose as seen in Mutant (50.71±0.13/1.49±0.03 µU/L/mg protein) and Lean (42.23±0.15/2.51±0.02 µU/L/mg protein) as compared to Controls which was more insulin responsive (41.11±0.09/13.97±0.25 µU/L/mg protein), similar to our published data on ‘Obese’ phenotype (WNIN/Ob) [20].

**Table 2:** Insulin Secretion assay.

Parameter	Mutant	Lean	Control
Basal glucose (5.5 mM)	50.71±0.13*	42.23±0.15	41.11±0.09
High glucose challenge (17.5 mM)	1.49±0.03*	2.51±0.02	13.97±0.25

Insulin secretion was measured from 100 Insulin-like cellular aggregates (ILCAs) formed from BM-MSCs. The data indicates hyperinsulinemia of the basal Insulin levels in Mutants. However, with high glucose challenge (17.5 mM), ILCAs from Mutants were non-responsive followed by Lean and Control. Insulin values have been expressed as µU/L/mg protein. Values have been represented as Mean ± SE (n = 6) from three independent experiments performed in duplicates.

Asteric (*) indicates significance at p<0.05 by ANOVA compared to Control.

## Discussion

In the present study we showed that BM-MSCs from Mutant rats portray features of obesity with IGT/IR/hyperinsulinemia (HI) vis-à-vis lean body mass, and higher fat mass as compared to its Lean and Control and mimic the disease state *in vivo* of pre-clinical/clinical T2D with IR seen in human subjects [25]. The present strain of Mutants showed an incomplete dominant mutation exhibiting three phenotypes - homozygous lean (+/+), heterozygous carrier (+/−) and homozygous obese (−/−) in a 1∶2:1 Mendelian ratio. All the three phenotypes could be identified phenotypically as early as 35 days by the presence of a ‘kinky tail’, a trait unique to the WNIN obese mutant rats that co-segregates with obese and carrier but not lean [19], suggesting for an early onset of degenerative-like changes/symptoms in these Mutants. Interestingly, these Mutants demonstrated most of the secondary complications associated with T2D such as onset of cataract, cancers, infertility, etc., making them a good animal model to study the pathophysiological state underlying obesity and/obesity-induced diabetes [19], [26]–[28].

Microenvironment/niche plays a pivotal role towards maintenance of cellular homeostasis, cellular integrity and functions. Yet, the niche may also induce pathologies by imposing aberrant function to the cellular components observed in diseased states such as diabetes [29], [30], myelomas [31], neoplastic cells [32] and bone loss associated with T1D [17] etc. Inflammation and inflammatory milieu are known to be the underlying mechanism(s) operating in these disease states. Our present data on BM-MSCs isolated from Mutant phenotype showed an upregulated expression of (i) inflammatory genes - IL-6 and TNFα (Figure 5A) and (ii) stress-related proteins TNFα and RL-77 (Figure 5B–F) with a proportional increase in circulating TBARS in plasma, suggestive of cellular stress and inflammatory condition in the progenitor pool, similar to studies reported with BM-MSCs from diabetic [33] and obese -diabetic subjects [34] advocating the role of BM-MSCs in disease pathology. IR is the chief abnormality underlying adult onset degenerative diseases like diabetes, cardiovascular diseases (CVD), hypertension etc., and there has been increasing evidence to suggest causative links between inflammation and development of IR [1], [3] akin to our data. It has also been shown that TNFα can induce IR in human skeletal muscle by altering the insulin signaling pathway providing a unique system for molecular dissection *in vitro* [35]. The physiological source of increased circulating levels of TNFα in T2D remains unclear; however it is believed to be a major cytokine involved in the exchange between adipose tissue and muscle with increased levels in obesity and T2D possibly contributing to IR in skeletal muscle [36].

Fat is an integral part of the BM microenvironment [37], [38] and marrow adipogenesis has shown to be an integral part of adipocyte homeostasis and appears to be the major contributor as a non-adipose origin for the increased risk of visceral adiposity [39], [40]. Further, reports of Gimble et al., [41] suggest that non-adipose derived adipocytes, such as BM-progenitors form an important contributor towards the increased visceral fat accumulation seen mostly in post-menopausal women who are at an increased cardiometabolic risk as compared to pre-menopausal women [42]. In similar lines we have demonstrated for an increased adipogenesis from BM-MSCs of Mutant rats (1.6–1.8 fold) as compared to Leans and Controls, examined as a phased gene expression (ESC→ MSC→ Pre-adipocyte→ Mature adipocyte). Substantiating these have been a sequalae of events, which were more noted from BM-MSCs of Mutants showing an increase in: a) recruitment of stem cells (Oct-4 and Sox-2) (Figure 4A), b) ‘S’ phase of cell cycle/BrdU incorporation/PDA (Figure 3A–F), c) CD90/CD29 positive cells (Figure 2A–F), d) induction of preadipocyte (Dact-1/Pref-1) (Figure 4B), and e) accelerated formation of mature adipocytes (increase in PPARγ2 (Figure 4C)) [43] reflecting for an obesogenic environment. It has been reported that HI increases the percentage of S+G2-M population as noted in the present study amongst Mutant Compared to Lean and Control (Figure 3B–D) [44], [45]. A higher tendency for adipocytes derived from BM to home among the visceral adipose depots have been reported by Gimble et al., [41] and Gambacciani et al., [46] and visceral adiposity is a known contributor to a range of secondary complications of T2D [47] attributed to its lipolytic/lipotoxic nature [48], [49]. This along with increased pro-inflammatory adipokine (IL-6 and TNFα) and decreased adiponectin secretion (causing decreased insulin sensitivity) [50], [51] have been a known predictor for T2D with IR. In similar lines, the observed increase in adipocyte formation among BM-MSCs (Mutant) vis-à-vis with an increased expression of IL-6 and TNFα (pro-inflammatory adipokine) underline for visceral fat accumulation [39] in these Mutant rats as compared to its Lean and Control [19] and correlate positively with obesity, IR and IGT.

Obese phenotype memory has also been appreciated in these Mutants with an upregulated expression in PPARγ2, inflammatory cytokine production (IL-6 and TNFα) and with a relative low leptin expression among BM-MSCs, compared to its Lean, which marks for the disease memory of tissue [18], [52] suggesting for visceral adiposity/visceral fat mass accumulation in these Mutants [19] **.** Supporting data such as, an increased expression of Dact-1 (a Wnt/β-catenin antagonist) and PPARγ2 (Figure 4B&C) and decreased TGFβ1 expression (Figure 4C) in BM-MSCs, does indicate for accelerated adipocytic induction with a proportional decrease in anti-osteoblastic recruitment, which underlines for early onset osteoporotic changes in Mutants [19] similar to humans [42]. In fact, PPARγ2 is not only a master transcription factor of adipogenesis, but also decides the progenitor commitment either for an adipocyte or an osteocyte formation [37], [43], since both adipocytes and osteocytes share a common progenitor [41] and maintenance of optimum adipocyte number in the BM is controlled by a balanced pro-adipocytic (increased activity of PPARγ2) and an anti-osteoblastic MSC allocation (decreased TGF-β/Bone Morphogenic Protein (BMP), Wnt/β-catenin, and Insulin growth factor-1 (IGF-I) signaling pathways), in addition to C/EPBα which is responsible for the maintenance of fully differentiated phenotype of adipocytes [53].

T2D accounts for approximately 90–95% of all existing diabetic cases and is a major health concern. Interestingly, we have shown an increase in the number of ILCAs obtained from BM-MSCs derived from Mutants, suggestive of obese/obese-diabetic environment similar to the findings of Rosen et al., [39] and Sun et al., [54]. In support of these, we have also demonstrated a significantly upregulated expression of insulin (hyperinsulinemic) and a decreased PDX-1, (a transcription factor necessary for pancreatic development and β-cell maturation) and GLUT-2 (pancreatic glucose transporter) (Figure 4F) implicating for a hyperglycemic environment [18], [34], [55]. Such an ectopic expression of these genes in BM-MSCs possibly can be attributed to body’s adaptive mechanism in the management of hyperglycemia, since HI (increased insulin levels in circulation) precedes onset of IR and T2D [25], [27]. Despite increase in the formation of ILCAs, the ability to respond to high glucose challenge was impaired from Mutant and Lean as compared to Control (Table 2). This phenomenon could be associated to IR in peripheral tissues [25], [42] with relatively insufficient secretion of insulin, resulting in pancreatic β-cell dysfunction with severity and progress of the disease/due to the blunting effects of hyperglycemia [56], or by feedback suppression for a prevailing HI [57]. Nevertheless, our present findings for an altered insulin secretion in ILCAs obtained from BM-derived of Mutant and to some extent in Lean, does suggest for an altered cellular milieu [19], similar to the pre-diabetic/T2D subjects with IR [25]. Keeping in view of the promise(s) of this Mutant model system in T2D, our studies are in progress to delineate the mechanism(s) underlining Obesity/IGT/HI/IR, which form important predisposing factors precipitating in several micro- and macro-vascular complications associated with chronic T2D.

### Conclusions

Given the ease of accessibility and availability of MSCs, our present study forms the basis to report for the first time, that MSCs could be explored as a feasible *in vitro* stem cell model system to portray disease memory *in situ* (altered milieu) of pre-diabetes/T2D, associated with Obesity, IGT, HI with IR, a major metabolic disorder of global concern.

## Materials and Methods

### Animals

This study was approved by the Institutional Animal Ethical Committee (IAEC Committee, NCLAS, NIN (ICMR), Hyderabad/Committee for the Purpose of Control and Supervision of Experiments on Animals (CPCSEA) (Regd. No. 154/1999/CPCSEA) and experiments were carried out as per the animal ethical norms.

Four months old male Mutant and Lean rats from WNIN/GR-Ob colony along with age-matched Controls (WNIN) were housed in cages, fed on regular rat chow and maintained under optimal ambience of temperature, light (12 hour dark/light cycles), oxygen, humidity and ventilation till sacrificed. All studies were performed under identical conditions for all the three phenotypes. Animals were subjected to a 16 h fasting prior to euthenization to normalize for the differences in feeding patterns.

### Anthropometric and Plasma Measurements

Anthropometric measurements such as body weights and adipose depot weights were assessed for all animals used for the study and represented as Mean ± SE (n = 6). Subcutaneous and visceral (intraperitoneal) adipose depots were dissected from the Mutant, Lean and Control phenotypes during sacrifice, weighed and represented as Mean ± SE gms total adipose depot weight (subcutaneous+visceral) as per published protocols [58].

Blood (2–3 ml) was collected by retro-orbital vein puncture from Mutant, Lean and Control rats in pro-coagulant Vacutainers (Genire Bio, Germany) and plasma was stored appropriately till analysis. Blood glucose was measured during blood collection with a glucometer by the glucose oxidase method using OneTouch Horizon glucose measurement strips (Johnson and Johnson Ltd, Milpitas, CA, USA) and values were represented as mg/dl. Plasma insulin was measured using High Range Rat Insulin ELISA Kit (Mercodia, Sweden) as per the manufacturer’s protocol and values were represented as µU/ml [20] from six animals performed in duplicates. IR indices such as fasting glucose to insulin ratio (FGIR), Homeostasis model of assessment for insulin resistance (HOMA-IR) and Quantitative Insulin Sensitivity Check Index (QUICKI) have been calculated using mathematical formulas FGIR = FPG/FPI; HOMA-IR = FPG×FPI/2430 and QUICKI = 1/[Log(FPG)+ Log(FPI)], where FPG is Fasting Plasma Glucose in mg/dl and FPI is Fasting Plasma Insulin in µU/ml as reported in literature [21].

Thiobarbituric acid reacting species (TBARS) as a measure of global oxidative stress/lipid peroxidation was estimated in plasma by spectrophotometric measurement of MDA released at 532 nM as per previously published protocol [59] and values were represented as Mean ± SE nM/ml from six animals performed in duplicates.

### Isolation and Expansion of BM-MSCs

Animals were euthanized by CO_2_ asphyxiation and femur bones were collected under sterile conditions in DMEM/F12 (GIBCO, USA) supplemented with antibiotics (Himedia Laboratories Pvt Ltd, India). BM-MSCs were isolated as per the previously published protocol [60]. Briefly, under sterile conditions metaphyseal ends of femur were cut and marrow was flushed using a 2 ml syringe into DMEM/F12 supplemented with antibiotics. The blood clots were then dispensed uniformly, washed and centrifuged (3–4 times) at 1800 rpm for 10 minutes. The pellet obtained was an enriched fraction of mononuclear cells which was treated with RBC lysis buffer (Tris-Cl+NH_4_Cl), washed and seeded at a density of 5–7×10^3^ cells/cm^2^ in DMEM/F12+10% FBS onto T_25_ culture flask (Corning, USA) in a humified chamber at 37°C and 5% CO_2_ and cultured till confluence. Cells were passaged every 5–6 days using 0.25% trypsin-EDTA (Sigma, USA) and a split ratio of 1∶2 was adopted. Viability of the cultures was routinely monitored by trypan blue exclusion (TBE) as well as by MTT assay [22].

### Proliferation Rate

#### Population doubling assay (PDA)

To compare the *in vitro* expansion rate, proliferation rates of MSCs from Mutant, Lean and Control phenotypes were determined by seeding at a density of 1×10^4^ cells at passage 3 in 35 mm dish (BD, San Jones, USA) and were maintained in DMEM/F12+10% FBS in a humified chamber at 37°C and 5% CO_2_ for a period of 24, 48, 72, and 96 hrs. Cells were trypsinised at the end of culture duration (24, 48, 72 and 96 hrs) and TB excluded cells were counted using Neubar’s Hemocytometer [22]. PDA has been represented as Mean ± SE (n = 6) (as multiples of 1×10^4^) from three independent experiments performed in duplicates as a function of time.

#### Bromodeoxy Uridine (BrdU) incorporation studies

To label the cells in S phase, the primary cultures of BM-MSCs (passage 3) were incubated overnight in growth medium containing 10 µM BrdU salt (a structural analogue of nucleotide Thymidine, that gets incorporated into dsDNA during ‘S’ phase in place of thymidine in actively proliferating cells) as per the published method [61]. Incorporated BrdU is immunostained with anti-BrdU antibody and proliferation index was calculated as Proliferation index = (No. of BrdU positive nuclei/Total number of nuclei) x100 per field and values have been represented as Mean ± SE (n = 6) from three independent experiments performed in duplicates.

### Immunostaining

MSCs from Mutant, Lean and Control phenotypes at passage 3 were seeded at a density of 1×10^4^ cells per coverslip, and after attaining the confluency (70%), cells were fixed with freshly prepared 4% paraformaldehyde, permeabilized using chilled 50% methanol (v/v in water), blocked with 4% horse serum and processed for immunolocalization similar to Kiran et al., [29]. Briefly, the cells were incubated in the primary antibody for Mouse monoclonal anti-STRO-1 (1∶200) (Millipore, USA), Mouse polyclonal anti-RL-77 (1∶200) (kind gift, Dr. Vasudevan Seshadri, National Centre for Cell Sciences, Pune, India), PE Hamster Anti-Rat/Mouse TNFα (1∶100) (BD Biosciences, USA) at room temperature for 1 hour. Similarly, for BrdU labeling, after fixation and permeabilization, DNA was denatured with 2N HCl containing 0.5% TritonX-100 and 0.5% tween-20 for 30 minutes at room temperature, followed by neutralization with 1 mg/ml sodium borohydride, blocked and incubated with primary mouse anti-BrdU antibody (1∶100) (Sigma, USA) for 1 hour at room temperature. After repeated washings, coverslips for anti-STRO-1, anti-RL-77 and anti-BrdU were incubated with goat anti-mouse Alexa 568 (1∶200) (Molecular Probes, USA) for 1 hour at 37°C. After washing with PBS, cells were counter stained and mounted with 4′,6-diamidino-2-phenylindole (DAPI) (Vector laboratories, Burlingame, USA). The specimens were visualized and images captured using 405, 488 and 561 lasers in Leica SP5 series Confocal Microscope (Leica Microsystems, Mannheim, Germany) at magnifications indicated using scale bars. Fluorescence intensities were quantified using Leica Advanced Fluorescence software (Leica Microsystems, Mannheim, Germany) from six random frames and values are represented as Mean ± SE relative fluorescence units (RFU) from three independent experiments performed in duplicates.

### Flow Cytometric Analysis

#### Cell cycle analysis

MSCs isolated from Mutant, Lean and Control phenotypes at passage 3 were analyzed for cell cycle. Briefly 1.0×10^6^ cells/ml were fixed in 75% ethanol, washed in PBS and incubated with propidium iodide (3 g/L) containing RNase. The percentage of cells in G0–G1, S and G2-M phases was calculated using 488 nm red laser fluorescence and analyzed in BD FACS AriaII (BD, San Jose, CA). Peak fluorescence was gated to discriminate aggregates. The cells were analyzed for cell cycle analysis using FACS Diva Software in FACS Aria II (BD, San Jose, CA) [22].

#### Surface marker characterization

Parallely, single cell suspension of 0.5–1.0×10^6^ cells/ml (passage 3) of phosphate buffered saline (Ca^+2^/Mg^+2^ free), were incubated in 1∶100 dilution at 4°C for 30 minutes in dark for fluorescence tagged primary antibodies - CD29-FITC (HA 2/5), CD90-PE (Ox-7), CD31-PE (TLD-3A12) and TNFα-PE (TN3-19.12) (BD Biosciences, USA). A total of 10,000 events were acquired in FACS AriaII and data were analyzed by using FACS Diva software. Values are represented as Mean ± SE median fluorescence intensity (MFI) (CD90, CD29 and CD31)/percentage positivity (TNFα) of cells carried out from three independent experiments performed in duplicates.

### Semi-quantitative Reverse Transcription Polymerase Chain Reaction

#### Gene expression for adipogenic/pancreatic/inflammatory markers

Isolation of RNA, synthesis of cDNA and gene amplification was carried out as per our published protocol [62]. Briefly, RNA was isolated from cultured MSCs (1.0×10^6^ cells at passage 3) using TriReagent (Sigma, USA). 4 µg of RNA was used for cDNA synthesis using Enhanced Avian Reverse Transcriptase Enzyme (eAMV-RT) (Sigma, USA) and 2 µl of cDNA template was amplified using gene sequences given in Table 3. 5 µl of the amplified products were electrophoresed on 1.2% ethidium bromide stained agarose gel, visualized in GelDoc (BioRad, Italy) and densities of amplicons reflecting quantity of the gene were measured as intensity per square mm using QuantityOne software (BioRad, Italy). Expression levels of genes were normalized against the house keeping gene β-actin and values from three independent experiments performed in duplicates have been indicated (Mean ± SE).

**Table 3:** Gene Sequences of Primers.

Group	Gene	Forward Primer	Reverse Primer	Product Size (bp)
Embryonic Markers	Oct-4	cggaagagaaagcggact	gccggttacagaaccaca	157
	Sox2	cggcaaccagaagaacag	tctcggtctccgacaaaa	167
Mesenchymal Markers	Dact-1	cttgccatctccaagcag	tgccctgtgggacactac	206
Preadipocyte Markers	Pref-1	attcgtcgacaagacctgca	ccaccagcctggtgagcacg	356
Adipogenic Transcriptional Factors	PPARγ2	tgacagtgacttggccatatt	gcagagggtgaaggctcata	266
	C/EBPα	gccaagaagtcggtggataa	ccttgaccaaggagctctca	232
	TGFβ1	gcctccgcatcccacctttg	cgggtgacttctttggcgt	350
Mature Adipocyte Markers	Leptin	cctgtggctttggtcctatctg	aggcaagctggtgaggatctg	244
	GLUT-4	agagtgcctgaaacc	ccctaagtattcaagttctg	114
	Adiponectin	gtcactgtccccaatgttcc	agaggcctggtccacatttt	360
Insulin Signaling Markers	IRS-1	accacttggaacgtcgtg	tggggtccactctctgtg	210
Cytokine/Chemokine Markers	IL-6	cagccagttgccttcttg	tgcatcatcgctgttcat	219
	TNFα	gcaaaccaccaagcagag	cggagaggaggctgactt	232
Pancreatic lineage markers	Insulin	caatcatagaccatcagcaagc	tttattcattgcagaggggtgg	212
	PDX-1	tacaaggacccgtgcgcatt	tcaagttgagcatcactgcc	451
	GLUT-2	ccacccagtttacaagctc	tgtaggcagtacgggtcctc	325
Housekeeping Gene	β-actin	tgtgatggtgggaatgggtcag	tttgatgtcacgcacgatttcc	498

Abbreviations:

Pref-1: Preadipocyte factor-1, PPARγ2: Peroxisome proliferator activator receptor gamma 2, C/EBPα: CCAAT/enhancer- binding protein alpha, TGFβ1: Transforming growth factor beta 1, AdipoQ: Adiponectin, GLUT-4: Glucose Transporter -4, IRS-1: Insulin receptor substrate -1, IL-6: Interleukin-6, TNFα: Tumor necrosis factor alpha, PDX-1: Pancreatic duodenum homeobox-1, GLUT-2: Glucose Transporter -2.

### Differentiation Potential of MSCs

#### Adipocyte formation

Confluent monolayer of MSCs (passage 3) were trypsinized, seeded at a density of 1.0×10^6^ cells per 35 mm dish and cultured in cell culture medium till confluence. MSCs were subjected to adipogenic induction, as per the manufacturer’s instructions (Chemicon, USA) [23]. At the end of 15 days, cells were fixed in 3% paraformaldehyde and stained with Oil-Red stain to visualize the formation of lipid-droplet filled adipocytes. Adipogenic induction potential was quantitated by counting the number of Oil-red stained cells (indicating mature adipocyte formation) from six random fields using ACT2U software under light microscope (Nikon, Japan) at magnifications indicated using scale bars and values have been represented as Mean ± SE percentage of oil red positive cells from three independent experiments performed in duplicates.

### Transdifferentiation Potential of MSCs

#### Pancreatic differentiation-ILCAs

MSCs from Mutant, Lean and Control phenotypes were committed for pancreatic differentiation using the three step protocol [24]. Briefly, 1.0–1.5×10^6^ cells were seeded onto 35 mm dishes to reach confluence in SFM-A (DMEM/F12 (1∶1) (17.5 mM glucose), 1% BSA, 1X (ITS) (5 mg/l insulin, 5 mg/l transferrin, 5 mg/l selenium) (Sigma, USA) and 50 µM β-mercaptoethanol) supplemented with 4 nM Activin A (Sigma, USA), 1 mM sodium butyrate (Sigma, USA), and cultured for 2 days. On the 3^rd^ day, MSCs were changed to medium containing SFM-A+0.3 mM Taurine and cultured for another 2 days and finally shifted to SFM-C containing DMEM/F12 (1∶1) with 17.5 mM glucose, 1.5% BSA, ITS, 3 mM Taurine (Sigma, USA), 100 nM Glucagon-Like Peptide (GLP)-1 (Sigma Aldrich), 1 mM Nicotinamide (Sigma, USA) and 1X Nonessential Amino Acids (NEAAs) (GIBCO, USA) on the 5th day and cultured till day 10. Integrity and specificity of ILCAs formed was assessed by staining with Dithizone (DTZ) (a sulfur containing organic compound, which binds to zinc ions in islet’s β-cells) [63].

### Insulin Secretion Assay

Insulin secretion assay (ISA) was performed to evaluate the functional response of ILCAs formed from Mutant, Lean and Control Phenotypes. Briefly, 100 ILCAs formed from MSCs were subjected to insulin secretion at basal (5.5 mmol/L) followed by high glucose challenge (17.5 mmol/L) in Krebs Ringer Bicarbonate (KRB) buffer. Insulin secreted into the buffer was estimated using High Range Rat Insulin ELISA Kit (Mercodia, Sweden) and values were represented as µU/L insulin/mg protein [20].

### Statistical Analyses

Descriptive statistics (Mean, SD) were calculated for all the samples and values have been represented as Mean ± SE. To compare between the phenotypes analysis of variance (ANOVA) followed by post hoc tests (LSD/Dunnett’s C test) were carried out based on Levene’s test for equality of error variances. p<0.05 was considered significant for all the tests. All the statistical analyses were performed using SPSS vs 15.0 software.
